# Mouse Middle Ear Ion Homeostasis Channels and Intercellular Junctions

**DOI:** 10.1371/journal.pone.0039004

**Published:** 2012-06-15

**Authors:** Lisa M. Morris, Jacqueline M. DeGagne, J. Beth Kempton, Frances Hausman, Dennis R. Trune

**Affiliations:** Oregon Hearing Research Center, Department of Otolaryngology – Head and Neck Surgery, Oregon Health and Science University, Portland, Oregon, United States of America; University of Minnesota Medical School, United States of America

## Abstract

**Hypothesis:**

The middle ear contains homeostatic mechanisms that control the movement of ions and fluids similar to those present in the inner ear, and are altered during inflammation.

**Background:**

The normal middle ear cavity is fluid-free and air-filled to allow for effective sound transmission. Within the inner ear, the regulation of fluid and ion movement is essential for normal auditory and vestibular function. The same ion and fluid channels active in the inner ear may have similar roles with fluid regulation in the middle ear.

**Methods:**

Middle and inner ears from BALB/c mice were processed for immunohistochemistry of 10 specific ion homeostasis factors to determine if similar transport and barrier mechanisms are present in the tympanic cavity. Examination also was made of BALB/c mice middle ears after transtympanic injection with heat-killed *Haemophilus influenza* to determine if these channels are impacted by inflammation.

**Results:**

The most prominent ion channels in the middle ear included aquaporins 1, 4 and 5, claudin 3, ENaC and Na^+^,K^+^-ATPase. Moderate staining was found for GJB2, KCNJ10 and KCNQ1. The inflamed middle ear epithelium showed increased staining due to expected cellular hypertrophy. Localization of ion channels was preserved within the inflamed middle ear epithelium.

**Conclusions:**

The middle ear epithelium is a dynamic environment with intrinsic mechanisms for the control of ion and water transport to keep the middle ear clear of fluids. Compromise of these processes during middle ear disease may underlie the accumulation of effusions and suggests they may be a therapeutic target for effusion control.

## Introduction

Otitis media is a common disorder affecting approximately 90% of children and is the leading cause of conductive hearing loss in children worldwide. [1] A fluid-free and air-filled middle ear cavity is required for effective sound transmission. Middle ear effusion is a component of acute otitis media. Although generally transient, 67% of children have persistent middle ear effusions up to 4 weeks after resolution of the acute infection and 25% can have an effusion present at 3 months. [2] Prolonged middle ear effusion and resultant conductive hearing loss can lead to language and developmental delay. Often surgical intervention is required to eliminate persistent effusions.

The natural mechanisms for clearing middle ear effusions include mucociliary transport, pumping action via the Eustachian tube, and transepithelial fluid transport. [3], [4], [5], [6], [7] It is this last method that is the least understood and the focus of this study. Transepithelial ion and water transport for endolymph homeostasis in the inner ear tightly controls the endolymphatic potential required for normal auditory and vestibular function. [8], [9], [10], [11] Some of these same channels have been reported for the middle ear epithelium and may play a similar role of fluid control within the middle ear space. [3], [12], [13], [14] However, a thorough evaluation of such ion and water transport channels in the middle ear has not been conducted. Therefore, the present study employed immunohistochemistry to identify the expression and localization of ion channels, water channels, and junctional proteins in the normal murine middle ear epithelium to determine if they are potentially involved in fluid regulation. An evaluation also was made of changes in the expression of these same channels and junctions during inflammation to determine if their altered expression could potentially contribute to uncontrolled middle ear effusion. A better understanding of these transport mechanisms may lead to improved therapeutic control of effusions that are common in otitis media.

## Materials and Methods

### Ethics Statement

All animal procedures in the study followed the guidelines for the care and use of laboratory animals of the national institutes of health and were approved by the Oregon Health and Science University Institutional Animal Care and Use Committee, Protocol # A149 (Dennis R. Trune). All surgery was performed under ketamine and xylazine anesthesia, and all efforts were made to minimize suffering.

Ten BALB/c mice (2–3 months of age) were screened for absence of middle ear fluid with otomicroscopy. Five normal BALB/c mice were anesthetized with a subcutaneous injection of ketamine (100 mg/ml; 0.067 mg/gm) and xylazine (20 mg/ml; 0.013 mg/gm), and intracardially perfused with 1 mL of saline, followed by 20 mL of fixative (3% paraformaldehyde in 0.1 M phosphate buffer). Following overnight immersion fixation, the bulla and inner ear were removed intact, microwave decalcified in 10% EDTA-Tris buffer solution, dehydrated through a graded series of alcohols and CitriSolv™ (Fischer Scientific Pittsburgh, PA), and embedded in paraffin. Specimens were then sectioned at 5 µm thickness, serially mounted on glass slides, deparaffinized, and rehydrated. Cross-sections containing both inner ear and middle ear structures were chosen for indirect immunohistochemistry with primary antibodies (Table 1) against aquaporins (AQP1, AQP4, AQP5), Na+,K+-ATPase α1, epithelial Na+ channel (ENaC), K^+^ channels (inward rectifier KCNJ10, voltage gated KCNQ1), tight junction claudins (3,4), and gap junction β2 connexin 26 (GJB2).

**Table 1:** Primary and Secondary Antibodies.

Primary Antibodies	Source	Dilution
Aquaporin 1Rabbit Affinity Purified Polyclonal Antibody	Millipore Corp. (Billerica, MA )Catalog # AB3065	1∶100
Aquaporin 4Rabbit Affinity Purified Polyclonal Antibody	Abcam Inc. (Cambridge,MA)Catalog # ab46182	1∶10
Aquaporin 5Rabbit Affinity Purified Polyclonal Antibody	Alpha Diagnostic Int. (San Antonio, TX)Catalog # AQP51-A	1∶10
Claudin 3Rabbit Affinity Purified Polyclonal Antibody	Abcam Inc. (Cambridge,MA)Catalog # ab15102	1∶10
Claudin 4Rabbit Affinity Purified Polyclonal Antibody	Imgenex Corp. (San Diego, CA)Catalog # IMG-80489	1∶10
αENaC (H-95)Rabbit Polyclonal Antibody	Santa Cruz Biotechnol. Inc. (Santa Cruz, CA)Catalog # sc-21012	1∶10
Gap Junction, Beta 2Rabbit Affinity Purified Polyclonal Antibody	Lifespan Bioscience (Seattle, WA)Catalog # LS-C14526	1∶10
KCNJ10, (C-terminal)Rabbit IgG Purified Polyclonal Antibody	Abcam Inc. (Cambridge, MA)Catalog # ab80959	1∶10
		
KCNQ1, (Carboxyterminal)Rabbit Affinity Purified Polyclonal Antibody	Abcam Inc. (Cambridge,MA)Catalog # ab77701	1∶10
Na+/K+ ATPase α-1Rabbit Affinity Purified Polyclonal Antibody	Upstate Biotechnology (Lake Placid, NY)Catalog # 06-520	1∶10
**Secondary Antibody**		
Alexa Fluor® 488 Goat Anti-Rabbit IgGAffinity Purified	Invitrogen (Carlsbad, CA)Catalog # A-11008	1∶50
**Diluent**		
Da Vinci Green Antibody Diluent	Biocare Medical, LLC (Concord, CA)Catalog # PD900	

Following binding by a secondary antibody conjugated to Alexa Fluor 488, sections were observed with either a Leica DMLB fluorescence microscope or BioRad MRC1024 laser confocal attachment on a Nikon TE300 inverted fluorescence microscope. Sections from five mice were stained for each antibody. Sections contained both inner ear and middle ear structures, with the former serving as the positive control to confirm staining of these known inner ear proteins. Each slide contained a negative control section that was subjected to identical methods but did not receive any primary antibody. The middle ear tissue was assessed for localization and relative degree of staining.

Inflammation was induced in the remaining five BALB/c mice utilizing the acute middle ear disease mouse model described previously. [15] Acute otitis media (OM) was created by bilateral transtympanic inoculation with heat-killed *Haemophilus influenza.* These mice were sacrificed 72 hours later and each bulla was harvested and processed as above.

## Results

### Normal Middle Ear Epithelium

Immunohistochemistry showed significant expression of 9 out of 10 channels and junctional proteins in the normal murine middle ear. Parallel localization of each of the 10 antibodies listed in Table 1 was confirmed for the inner ear positive controls. A summary of the findings for inner ear and middle ear structures was developed based on consistent and specific antibody staining (Table 2).

**Table 2:** Comparison of Inner Ear and Middle Ear Antibody Staining.

ANTIBODY	INNER EAR	NORMAL MIDDLE EAR	INFLAMED MIDDLE EAR
**Aquaporin 1**	Lining of scala tympani Lining of semicircular canal Crista ampullaris Type III fibrocytes of spiral ligament	Submucosa of ME epithelium Submucosaof TM Submucosa of round windowmembrane	Submucosa of ME epithelium Submucosa of TM Submucosa of round window membrane
**Aquaporin 4**	Spiral ganglion Organ of Corti Bone of otic capsule Lining of scala tympani Liningof scala vestibuli	ME epithelium, cytoplasm Boneof ossicles	ME epithelium, cytoplasm Mucosal layer of TM, inclusion bodies Epithelium on lateral (ME) surfaceof round window membrane
**Aquaporin 5**	Organ of Corti	ME epithelium, apical surface	ME epithelium, apical surface
**Claudin 3**	Marginal cells of stria vascularis,apical surface Reissner’s membrane Reticular membrane Spiral ganglion Organ of Corti Maculae, apical surface Periosteal lining ofotic capsule	ME epithelium, basal and lateralsurfaces	Basal and lateral cell walls Mucosal layer of TM, inclusion bodies
**Claudin 4**	Spiral ganglion Organ of Corti	No significant staining	No significant staining of ME epithelium Mucosal layer of TM, inclusion bodies
**Gap Junction** **β2**	Organ of Corti Spiral ligament Spiral limbus Lining of utricle Lining of saccule Macula,apical surface Lining of semicircular canals	ME epithelium, cytoplasm Epitheliallayer of TM	ME epithelium, cytoplasm Mucosal layer of TM, inclusion bodies Epithelium covering promontory
**ENaC**	Organ of Corti Macula, apical surfaceFibrocytes of spiral ligament Reissner’s membrane Spiral ganglion	ME epithelium, cytoplasm	ME epithelium, cytoplasm Mucosal and epithelial surface of TM Epithelium covering promontory
**Na^+^/K^+^-ATPase**	Stria vascularis Organ of Corti Spiralligament fibrocytes (Type II & IV) Spiralganglion Reissner’s membrane	ME epithelium, cytoplasm	ME epithelium, cytoplasm Mucosal layer of TM
**KCNQ1**	Marginal cells of stria vascularisVestibular dark cells	ME epithelium, cytoplasm	ME epithelium, cytoplasmMucosal and epithelial surface of TM
**KCNJ10**	Intermediate cells of stria vascularisOrgan of Corti	ME epithelium, cytoplasm	Mucosal and epithelial surface of TM

TM: tympanic membrane; ME: middle ear.

#### Aquaporins

**Figure 1:**
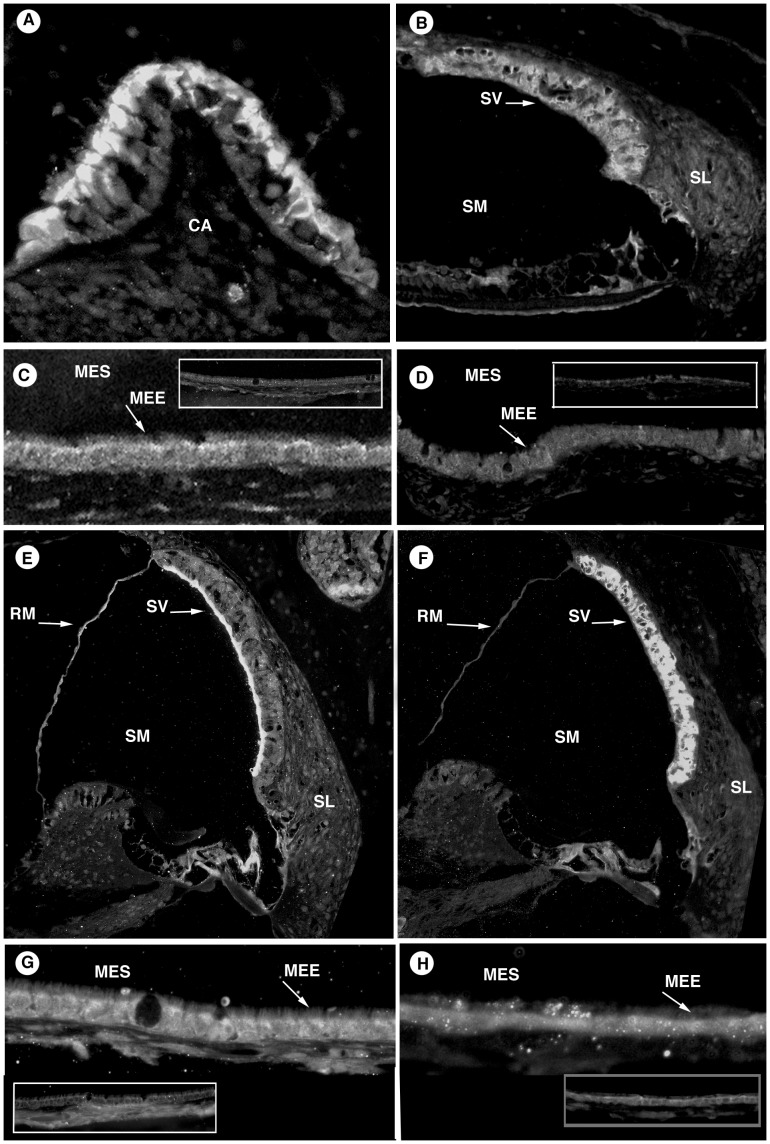
Aquaporins. Aquaporins 1, 4, and 5 in the inner ear (A-C) and middle ear (D-G). A: AQP1 in the inner ear showed positive staining of the lateral lining of the spiral ligament and some fibrocytes within the spiral ligament. B: AQP4 in the inner ear positively stained bone of the otic capsule, organ of Corti, and spiral ganglion. C: AQP5 showed mild labeling of the inner ear organ of Corti. D: AQP 1 antibody stained the middle ear epithelial submucosa and capillary endothelium. E and F: Middle ear AQP4 labeled the middle ear epithelium itself and portions of the ossicular bones, malleus and incus. G: AQP5 in the middle ear appeared limited to the apical membranes of the epithelium. Negative controls are displayed as inset pictures. [SV, stria vascularis; SM, scala media; SL, spiral ligament; RM, Reissner’s membrane; OC, organ of Corti; ST scala tympani; MEE, middle ear epithelium; SUB, submucosa; MES, middle ear space; M, malleus; I, incus].

Aquaporin 1, 4 and 5 showed specific cellular localization with minimal overlap of their regional expression (Fig 1). Aquaporin 1 strongly stained the submucosa of the middle ear epithelium and within the capillary endothelium (Fig 1D). The inner ear positive control (Fig 1A) showed localization to type III fibrocytes in the spiral ligament, the lining of the scala tympani and semicircular canals, Reissner’s membrane and the round window membrane. Aquaporin 4 showed diffuse staining of the middle ear epithelial cells, as well as the ossicular bones (Figs 1E,F). Staining in the inner ear (Fig 1B) was limited to the spiral ganglion, organ of Corti, and bone of the otic capsule adjacent to the spiral ligament. Aquaporin 5 strongly localized to the middle ear mucosa, labeling the apical surface of ciliated, non-ciliated and basal cells (Fig 1G). In the inner ear this localized only faintly to the organ of Corti (Fig 1C).

#### Junctional complexes

**Figure 2:**
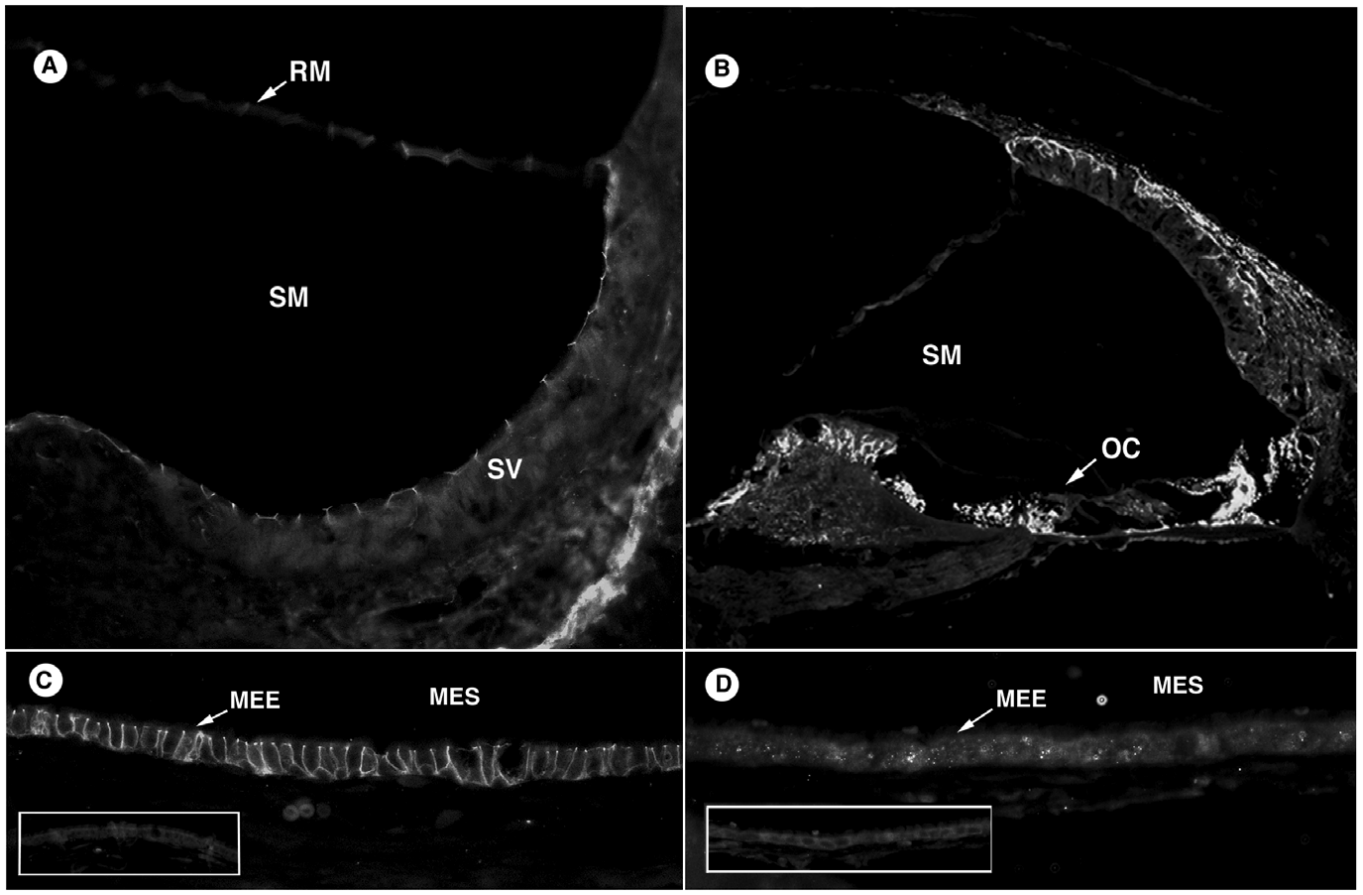
Junctional Complexes. A: Claudin 3 in the inner ear localized to the organ of Corti, Reissner’s membrane, periosteal lining of otic capsule, and the apical surface of the marginal cells of stria vascularis. B: GJB2 in the inner ear positively stained between fibrocytes in the spiral ligament, as well as cells within the organ of Corti and spiral limbus. C: Claudin 3 in the middle ear occurred in the intercellular junctions along lateral and basal cell membranes in the mucosal epithelium. D: GJB2 in the middle ear also stained the middle ear epithelium. Negative controls are displayed as inset picture. [SV, stria vascularis; SM, scala media; RM, Reissner’s membrane; OC, organ of Corti; MEE, middle ear epithelium; MES, middle ear space].

In the inner ear (Fig 2A) claudin 3 localized to the organ of Corti, spiral ganglion, Reissner’s membrane, apical surface of marginal cells of the stria vascularis, apical surface of the crista ampullaris and periosteal lining of the otic capsule. Claudin 3 (Fig 2C) in the middle ear localized to the intercellular space at the lateral and basal surfaces of the epithelial cells, with strong immunoreactivity of all cell types (Fig 2C). Claudin 4 was not consistently expressed in the middle ear and showed weak staining of the cytoplasm of epithelial cells (data not shown). GJB2 in the inner ear controls (Fig 2B) demonstrated strong localization between fibrocytes of the spiral ligament, organ of Corti, spiral limbus, linings of the vestibular organs, and the apical surface of the macula. It showed mild staining of the cytoplasm of the middle ear epithelia (Fig 2D), as well as strongly localizing to the epithelial surface of the tympanic membrane.

#### Ion channels

**Figure 3:**
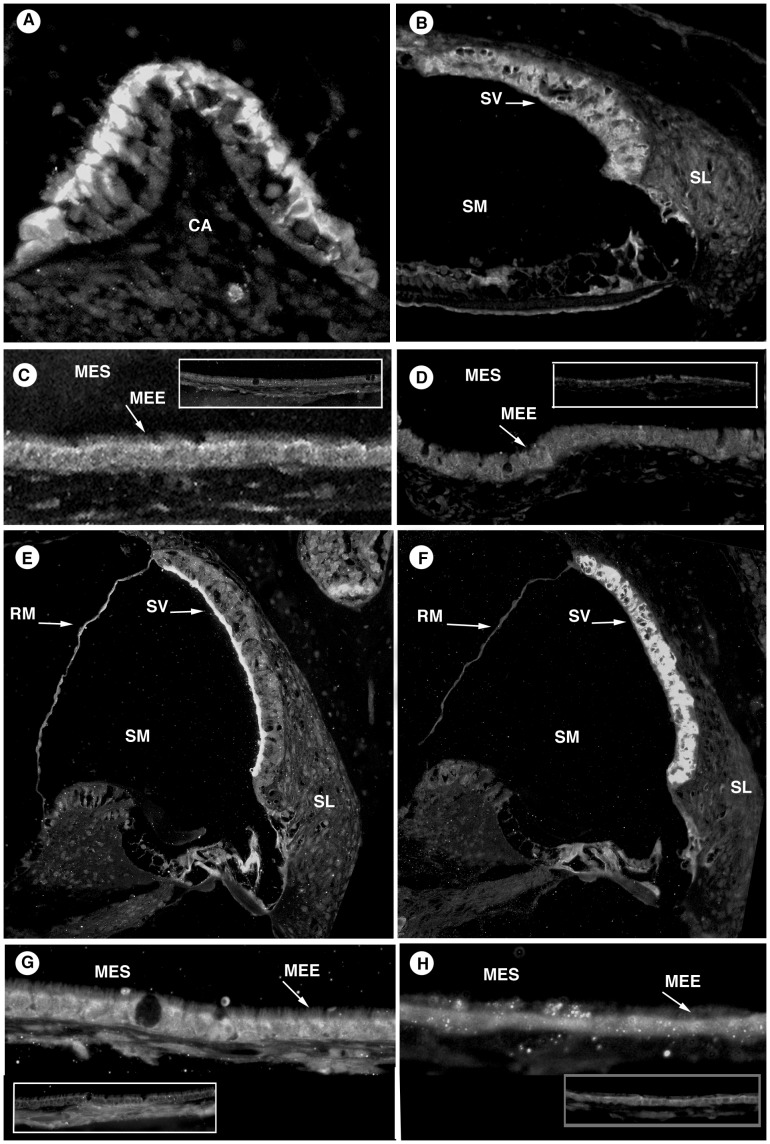
Ion Channels. A: ENaC in the inner ear occurred in the apical membrane of the crista ampullaris, as well as other places. B: Na+,K+-ATPase staining in the inner ear stained the stria vascularis, fibrocytes of the spiral ligament, and the organ of Corti. C: ENaC in the middle ear was located in the apical membrane of the epithelium. D: Na^+^,K^+^-ATPase middle ear diffusely stained the lining epithelium. E: KCNQ1 of the inner ear occurred at the apical surface of marginal cells of stria vascularis. F: KCNJ10 in the inner ear was seen, staining the intermediate cells of stria vascularis and organ of Corti. G: KCNQ1 stained the middle ear epithelium. H: KCNJ10 also stained the middle ear epithelium. Negative controls are displayed as inset pictures. [CA, crista ampullaris; SV, stria vascularis; SM, scala media; SL, spiral limbus; RM, Reissner’s membrane; OC, organ of Corti; MEE, middle ear epithelium; MES, middle ear space].

ENaC in the inner ear (Fig 3A) localized to the organ of Corti, fibrocytes of the spiral ligament, spiral ganglion, Reissner’s membrane, and the apical surfaces of the maculae and cristae ampullaris. ENaC showed diffuse staining of the cytoplasm of the middle ear epithelial cells with predominance at the apical surface (Fig 3C). Inner ear Na^+^,K^+^-ATPase (Fig 3B) was positive in the stria vascularis, type II and IV fibrocyte regions of the spiral ligament, spiral ganglion, organ of Corti and Reissner’s membrane. The antibody to Na^+^,K^+^-ATPase stained the cytoplasm of the middle ear epithelia cells (Fig 3D). In the inner ear, KCNQ1 strongly stained the apical surface of the marginal cells of stria vascularis (Fig 3E), while KCNJ10 localized to the intermediate cells of the stria vascularis and to supporting cells in the organ of Corti (Fig 3F). The potassium channels KCNQ1 and KCNJ10 localized to the cytoplasm of the epithelial cell in the middle ear (Figs 3G,H).

### Inflamed Middle Ear Epithelium

**Figure 4:**
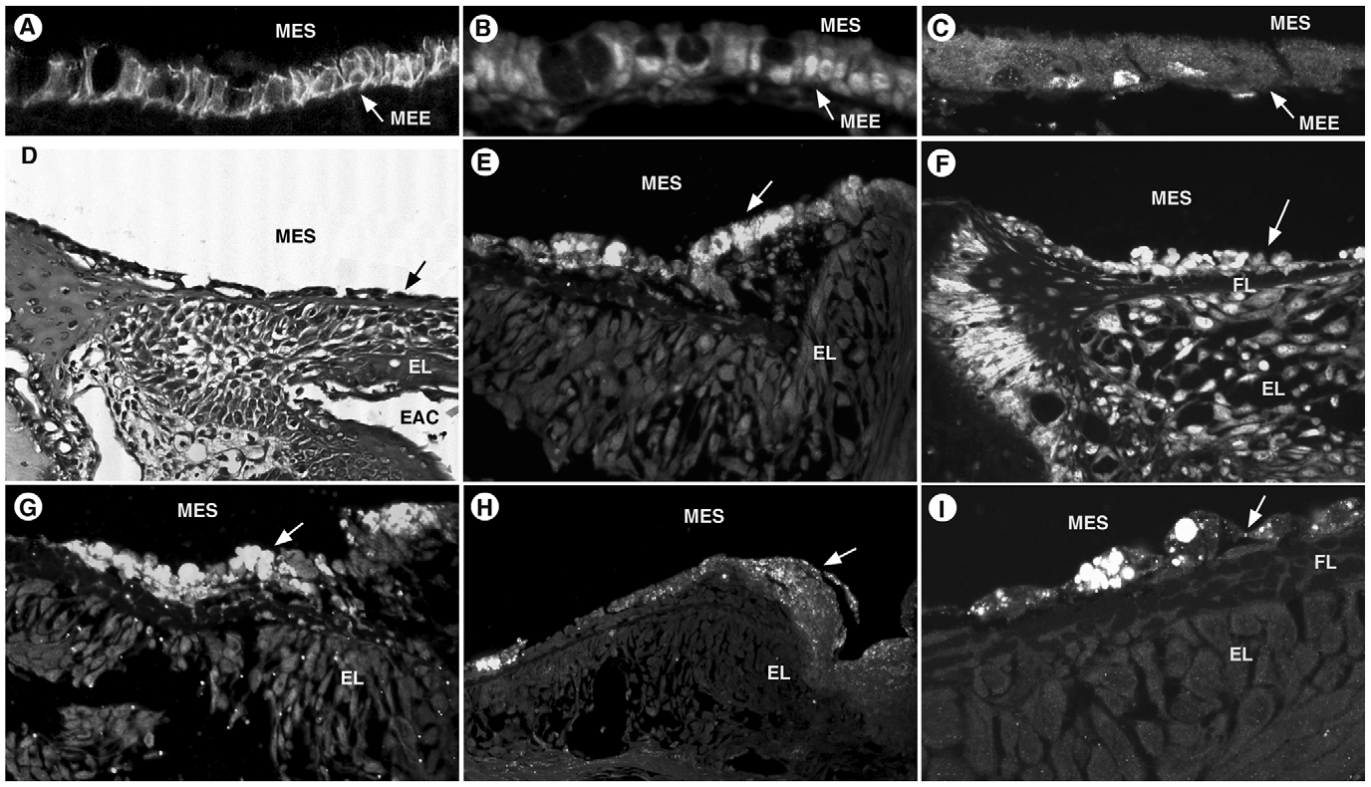
Inflamed Middle Ear Epithelium. Figures A-C show hypertrophied mucosa with increased number of secretory cells. Immunolocalization is similar to the normal middle ear epithelium. A: Claudin 3, middle ear mucosa. B: KCNQ1, middle ear mucosa. C: ENaC, middle ear mucosa. D: Inflamed TM under brightfield microscopy, demonstrates the 3 cell layers of the TM (mucosal layer, fibrous layer and epithelial layer) and the hyperplasia and edema that occurs with inflammation. Figures E-I show changes occurring at the TM during inflammation. E: Na^+^,K^+^-ATPase demonstrates increased reactivity on the mucosal surface of the TM. F: KCNQ1 shows strong reactivity on both the epithelial and mucosal surfaces of the TM with no reactivity of the fibrous central layer. G: AQP4, strong staining occurred on the mucosal surface of the TM. H: GJB2 stained on the mucosal surface with no staining of the hyperplastic epithelial layer, while in the non-inflamed middle ear the TM stained only on the epithelial surface. I: Claudin 4 had no staining of the middle epithelium in non-inflamed ears, however the mucosal surface was very reactive in the setting of inflammation. On high magnification the reactive components appeared as well-circumscribed inclusion bodies within the cytoplasm of the mucosal epithelium. [MEE, middle ear epithelium; MES, middle ear space; ML, mucosal layer of TM; FL, fibrous layer of TM; EL, epithelial layer of TM; EAC, external auditory canal].

All of the previously described channels and junctions stained in the normal middle ear epithelium, except for claudin 4, while all 10 primary antibodies stained in the inflamed middle ear epithelium. Generally, the inflamed mucosa showed increased strength of staining reflecting the proliferation of mucosa and increased secretory cells (Fig 4A–C) in response to bacteria, especially in the TM (Fig 4D–I). The localization of channels remained similar between normal and inflamed middle ear epithelium, except at the TM. With the presence of inflammation, AQP 4, claudin 4, GJB2, ENaC and Na^+^,K^+^-ATPase showed greater staining on the mucosal surface of the TM. Some primary antibodies showed reactivity to only the mucosal surface of the TM (AQP5, claudin 3 and 4, and GJB2) while others had reactivity of both the mucosal and epithelial surfaces of the TM (AQ4, KCNJ10, KCNQ1, ENaC and Na^+^,K^+^-ATPase).

#### Aquaporins

With AQP1 and AQP5 there were no changes to expression or localization in the inflamed epithelium compared to normal epithelium. The inflamed TM is thickened and edematous, but AQP1 continues to be localized to the submucosa of both the mucosal and epithelial surfaces. AQP5 localizes to the apical surface of middle ear mucosa and TM in both normal and inflamed ears. AQP4 was found throughout the cytoplasm in the normal middle ear and was more uniform throughout the cytoplasm of the inflamed middle ear epithelium. In the normal TM the outer epithelial layer was more reactive, however in the inflamed TM the mucosal layer is more reactive (Fig 4G).

#### Junctional proteins

Claudin 3 stained strongly throughout all middle ear mucosa and there appeared to be no change in it’s the location in inflamed mucosa. However, these tight junctions were more abundant as the epithelium proliferated (Fig 4A). The inflamed tympanic membrane showed reactivity of its mucosal surface, but not the epithelial surface. Claudin 4, which did not show significant staining of middle ear epithelium in either normal or inflamed specimens, did strongly stain the mucosal surface of the TM in the inflamed middle ears. This staining was localized to inclusion bodies within the epithelial cells on the mucosal lining of the TM (Fig 4I). Gap Junction staining was seen only in a small amount in the cytoplasm of the middle ear epithelium of both normal and inflamed ears. In the normal TM it was strongly reactive in the epithelial layer, but was most reactive in the hypertrophic mucosal layer during inflammation (Fig 4H).

#### Ion channels

Each of the ion transport channels was found to be present diffusely within the cytoplasm of middle ear mucosa of both normal and inflamed ears. In the inflamed middle ear mucosa, ENaC was not as prominent on the apical surface (Fig 4C) as it was in the normal mucosa. The inflamed TMs were found to be immunoreactive on both the mucosal and epithelial surfaces with the mucosal surface having increased reactivity for ENaC and Na^+^,K^+^-ATPase (Fig 4E).

## Discussion

The results of this study reveal multiple ion and water regulatory channels and junctions are present within in the normal middle ear epithelium. These same ion homeostasis channels have been extensively studied within the inner ear. [8], [9], [10], [11] They orchestrate the delicate balance of ion composition that must be preserved between the endolymph and perilymph to maintain the endocochlear potential. Multiple hearing disorders result from defects in these ion and water transport mechanisms, [8], [9], [10], [11] implying that similar disruptions of fluid balance could occur in the middle ear.

Fluid and debris are continually being cleared from the middle ear cavity by mucociliary clearance. This is accomplished by a specialized two-layer mucus blanket that lines the middle ear epithelium: a superficial layer traps debris and foreign particles and a deep periciliary layer that has high water content. [16], [17] The fluidity of the periciliary layer allows the cilia to move freely and proper mucociliary function requires tight regulation of the composition of this fluid. Transepithelial fluid transport via homeostatic ion and water channels may play a role in regulating this periciliary layer, aiding in mucociliary clearance. Variations in periciliary layer thickness occur in children with OME [5] which may represent an underlying dysfunction in the homeostatic mechanism. In addition to maintenance of the periciliary layer, transepithelial fluid transport plays a significant role in clearing middle ear effusions. Physiologic studies have shown that the epithelial sodium channel (ENaC) and aquaporin channels control fluid absorption in the middle ear. The fluid absorptive function of the middle ear epithelium is largely dependent upon the apical surface amiloride-sensitive ENaC. [3], [12], [13], [18] ENaC probably is the rate-limiting absorption channel, with other channels contributing to the ion and fluid homeostasis. Thus, ENaC and aquaporin channels play the major role in regulating the periciliary layer in the middle ear to allow for efficient mucociliary transport to occur, with their dysfunction affecting not only transepithelial fluid transport but also mucociliary transport.

Aquaporins (AQP) are a family of transmembrane water-permeable channels that allow free passage of water down an osmotic gradient. To date, there are 14 subtypes of aquaporins in mammals that are vital to proper functioning of many organs, especially the inner ear. [19], [20], [21], [22] Each subtype localizes to specific regions of the inner ear that generally do not overlap, creating highly regulated water pathways. [21], [22] In the middle ear, we found this region-specific localization to be maintained. AQP1 is localized to the submucosa and capillary endothelial cells, AQP4 in the cytoplasm, and AQP5 in the apical membrane of the middle ear mucosa. This is consistent with other studies of rats and guinea pigs showing similar distributions within the middle ear. [23], [24] These studies reported AQP4 was localized to the lateral and basal membranes of ciliated cells. However, our results did not show this localization, but rather found diffuse staining of cytoplasm of the entire epithelial cell, both ciliated and non-ciliated. There has also been controversy with localization of AQP 5. Kang et al. [24] demonstrated expression only on the apical surface of serous gland cells, while Minami et al. [23] found expression on the apical portion of ciliated, flat and columnar cells of rat middle ear epithelium. Our findings of AQP 5 expression in mouse middle ear epithelium were consistent with Minami et al. [23] The specific localization of each aquaporin subtype to a different region of the middle ear mucosa indicates that they are involved in an intricate and coordinated mechanism of water homeostasis.

Claudins are tight junction proteins which form intercellular barriers to fluid transport and regulate the paracellular pathway. There are over 24 types of claudins described, with eleven varieties found in the inner ear. [25], [26] In this study claudin 3 stained avidly within the tissue of both the inner and middle ear. Claudin 4 was present within the organ of Corti of a few animals, but was not found to have significant staining of the middle ear. Previous studies have found claudin 4 to be present in the marginal cells of the stria vascularis in guinea pigs. [26] However, another study looking at the inner ears of mice did not find claudin 4 to be expressed. [25] The abundant middle ear staining of claudin 3 in the present study suggests that it is needed to maintain a tight seal between epithelial cells. This water barrier may contribute to the prolonged presence of an effusion in some children.

Gap junctions are specialized intercellular connections that allow free passage of specific ions and small molecules between the cytoplasm of adjacent cells. These proteins play a vital role in the cochlea maintaining the K^+^ recycling pathway that generates the endocochlear potential. The most common hereditary cause of hearing loss is a mutation of GJB2. [9], [10], [27] In this study, GJB2 was found within the middle ear epithelial cytoplasm but weakly stained. Interestingly, it was also found to strongly stain the tympanic membrane, but changed localization from the epithelial surface to the mucosal surface in the setting of inflammation. It is unclear what role gap junctions play in middle ear homeostasis.

During acute inflammation of the middle ear mucosa, fluid accumulation occurs within the middle ear cavity. A recent study has shown that gene expression for the ion and water channels is either downregulated (AQP 1,5, and Na+,K+-ATPase) or unchanged (ENaC, GJB2, KCNQ1 and KCNJ10) during acute inflammation, but the junctional complexes (claudin 3 and 4) are upregulated. [28] This indicates that extracellular fluid accumulation may be due to dysfunction of the transepithelial ion and fluid channels within the middle ear epithelium, tipping the homeostatic balance towards middle ear effusion. The upregulation of the tight junctions (claudin 3 and 4) form water barriers between cells and therefore blocks egress of fluid out of the middle ear cavity.

On the protein level, this study demonstrates the presence and varied locations of these channels in the normal middle ear epithelium. It also demonstrates that the ion and fluid channels are still present and their locations are maintained during acute inflammation. Many of the changes found during the acute inflammatory phase occurred at the level of the tympanic membrane. Interestingly aquaporin 4, claudin 4 and gap junction β2 developed strongly staining inclusion bodies within the mucosal layer of the tympanic membrane. The role of changes occurring at the TM is unknown and may represent physiologic changes associated with inflammation and repair. Further studies are needed to better evaluate these inclusion bodies to determine if they have a functional role in effusion regulation.

To our knowledge this is the first comprehensive study of ion and water homeostasis channels within the middle ear. The presence of these channels within the middle ear epithelium suggests the middle ear is a tightly controlled environment for the regulation of fluid movement. In the setting of inflammation these proteins maintained their localization within the middle ear mucosa with the main changes occurring at the TM. It is anticipated that as future studies identify the roles of these homeostasis mechanisms in fluid control, a more comprehensive mechanism of middle ear effusion control will be identified. This may lead to interventional treatments that will reduce effusions and limit the morbidity of otitis media.

### Conclusion

Fluid and ion homeostasis within the middle ear is controlled by a series of channels and junctions that are well described to tightly control fluid and ion composition within the inner ear. While the mechanism of fluid clearance in the middle ear is poorly understood, the distinctive locations of many of these transporters, especially the aquaporins, suggest that middle ear fluid is regulated by a coordinated process. These findings also suggest that ion homeostasis channels may be responsible for maintaining the appropriate composition and volume of the periciliary layer. Disruption of any of these channels or transporters may be the mechanism of fluid accumulation and persistence in otitis media. Further study is needed to better understand these transport processes that may guide targeted therapy to prevent or treat middle ear effusions, therefore preventing numerous surgeries and prolonged conductive hearing loss.
