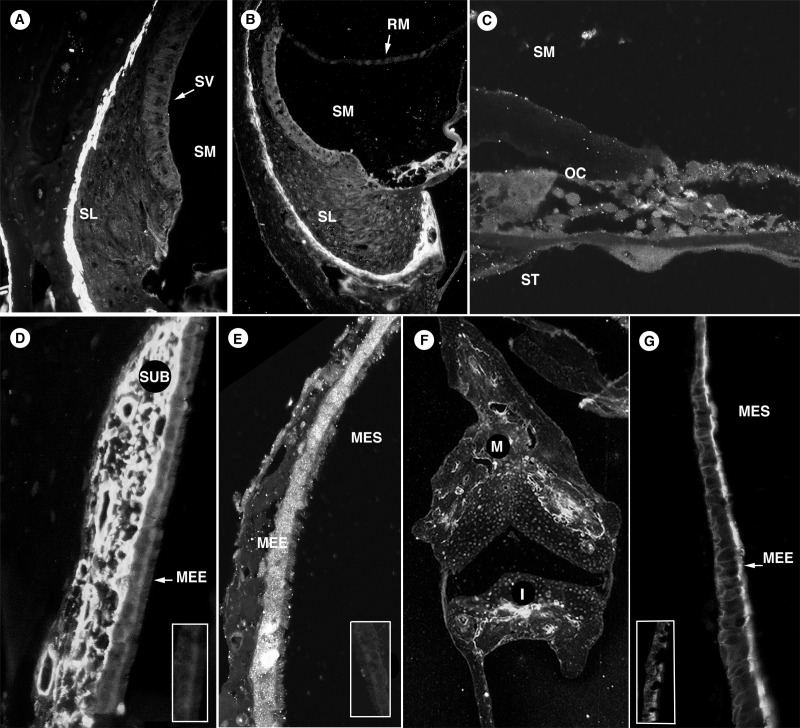# Correction: Mouse Middle Ear Ion Homeostasis Channels and Intercellular Junctions

**DOI:** 10.1371/annotation/2d2174c8-9c7a-4afd-9b0b-d9e96cfcb009

**Published:** 2013-04-01

**Authors:** Lisa M. Morris, Jacqueline M. DeGagne, J. Beth Kempton, Frances Hausman, Dennis R. Trune

The version of Figure 1 that exists in the article is incorrect. The correct version can be found here: 

**Figure:**